# Supplementary material for: “So, if she wasn’t aware of it, then how would everybody else out there be aware of it?”—Key Stakeholder Perspectives on the Initial Implementation of Self-Collection in Australia’s Cervical Screening Program: A Qualitative Study
Source: Int J Environ Res Public Health. 2022 Nov 27;19(23):15776. doi: 10.3390/ijerph192315776 (PMC9739016; doi:10.3390/ijerph192315776)
Supplement: Supplementary file 1 [file ijerph-19-15776-s001.zip › ijerph-2004341-supplementary.pdf]

Contents

Section S1 – Key stakeholder interview guidelines – general 1

Section S2 – Proctor Implementation Outcomes Framework 5

Section S1 – Key stakeholder interview guidelines – general

Rapport building

Introduce yourself

Introduce the study: On December 1st 2017, a new approach to cervical screening was implemented in Australia. In the same year, the National Health and Medical Research Council (NHMRC) funded a new Centre for Research Excellence (CRE) in Cervical Cancer Control which is evaluating new cervical screening approaches in the context of HPV vaccination.

What we plan to do and why: As a part of the planned CRE activities, we are interviewing a range of program partners and stakeholders such as yourself, to document the opportunities and challenges they are experiencing as the new National Cervical Screening Program is implemented. The interview will take around 30 minutes of your time. I would like to record this interview with your permission. The interview will be transcribed and then analysed, however, you will not be identified in any transcription or publications that arise from this.

Do you have any questions before we begin?

Interview guideline

1. Please can you tell me a little bit about your current, or previous roles, as they relate to the cervical screening program?

2. Overall, how acceptable do you think the changes to the program are to screening providers and program partners?

   {PROBES, if needed}

   How acceptable do you think providers and partners find:

- Delayed starting age for screening (25+ years) and later exit age
- Increased screening interval
- New screening test (HPV test not cotest or cytology alone)
- Sample collection
- Self-collection
- Management pathways
- New national register
- Colposcopy reporting

3. How acceptable do you think the changes are to women?

   {PROBES, if needed}

   How do you think women have responded to:

   - Delayed starting age for screening (25+ years)
   - Increased screening interval
   - New screening test
   - Self-collection

4. Do you think there are any differences in how the program should be implemented to meet the needs of Aboriginal and Torres Strait Islander women and women from culturally and linguistically diverse women? In your experience, is the program meeting their needs so far?

5. Which of the changes to the program have had the greatest impact (either positive or negative) on you and your organisation and in what ways?

   {see SPECIFIC PROBES per partner group }

6. Has implementing the new program had any cost impacts, either positive or negative, on you/your organisation? In what way? Were any costs incurred due to the delay in implementing the new program?

   {PROBES, if needed}

   For example, costs relating to workforce changes, IT changes, registry changes, branding/information changes, changes in revenue/billing arrangements

7. Do you think the new program has been successfully implemented so far (or as intended)?

   {PROBES if needed}

- Participation
- National register set up
- Resource availability

- Education and training
- Community/provider awareness
- Waiting times for colposcopy
- Laboratory services
- Accuracy of lab recommendations
- Guidelines being followed correctly

8. Is there anything that you think should or could have been done differently, in terms of program implementation?

   {PROBES if needed}
   What do you think of:
   - Consultation processes
   - Stakeholder and partner engagement
   - Governance arrangements
   - Transparency of processes
   - Sufficiency of government funding for implementation
   - Timing of education and training for providers
   - Timing of communications to providers and women
   - Timing of registry implementation
   - Timing of availability of self-collection
   - National guidelines for labs to process HPV tests
   - The new clinical guidelines
   - Handling of workforce issues (lab and colposcopy staffing)

9. Overall, what would you say is **<u>the most important factor</u>** in ensuring the successful implementation of the new program (eg what will make or break the program if it's not achieved)? Why is that?

10. Do you think there are any other factors that will facilitate the successful implementation of the new program?

11. Overall, what do you think is **<u>the biggest barrier</u>** to successful implementation of the new program (eg what do we need to overcome to succeed)? Why is that?

12. Do you think there are any other factors that will be barriers to successful implementation of the new program?

13. At a high level, what is **<u>your opinion</u>** of the changes to the program in totality?

14. What do **<u>you</u>** think are the short, medium and long term implications of the changes to the cervical screening program?

15. Is there anything else about implementing the new program that you would like to tell me about?

Section S2 – Proctor Implementation Outcomes Framework

Implementation outcomes	Level of analysis	Theoretical basis	Definition	Other terms in the literature	Implementation stage	Key research question(s)	Anticipated themes & subthemes
Acceptability	Individual provider, Individual consumer	Rogers: "complexity" and to a certain extent "relative advantage"	** Perception among implementation stakeholders that a given treatment, service, practice or innovation is agreeable, palatable or satisfactory* **Captures acceptability of the evidence based changes to cervical screening program among different stakeholders*	Satisfaction with various aspects of the innovation (e.g. **content, complexity, comfort, delivery, and credibility**)	Early, ongoing and later<br><br>**This is dynamic and changes with the stage of implementation*	1) How acceptable do you think are these changes (in the renewed program) to screening providers and other stakeholders?<br><br>2) How acceptable do you think are these changes (in the renewed program) to women/Indigenous women?	**ACCEPTABILITY**<br>A) Delayed starting age for screening (25+ years)<br>B) Increased screening interval (5 years)<br>C) New screening test & sample collection (liquid based sample & HPV based)<br>D) Self-collection (new pathway)<br>E) New referral and management pathways<br>F) New national register<br>G) Colposcopy reporting<br>H) Co-testing versus screening

Implementation outcomes	Level of analysis	Theoretical basis	Definition	Other terms in the literature	Implementation stage	Key research question(s)	Anticipated themes & subthemes
Adoption	Individual provider, Organisation, setting	RE-AIM: "adoption"<br>Rogers: "trialability" (particularly for early adopters)	*Intention, initial decision, or action to try or employ an innovation or evidence-based practice*	Uptake, utilization, initial implementation, intention to try	Early to mid (6, 12 or 18 months after implementation)	1) Can you please describe some of the changes in the renewed program that have impacted<br>-You (as a provider)<br>-Patients/clients<br>-Organisation<br>-Overall program<br>In what ways have they impacted you?<br><br>2) As a provider/organisation/ program, how are you coping with these changes?<br><br>3) Are you working with any Indigenous Australians or culturally or linguistically diverse groups for cervical screening?<br><br>4) If yes, are there any particular issues around cervical screening that may be more challenging? If so,	**<u>BARRIERS/UNINTENDED CONSEQUENCES/COPING STRATEGIES</u>**<br>**A) Lab perspective:** Workforce, processing time, infrastructure, business model, training/education/resources, 2016 NHMRC guidelines, new performance measures, registry support and operations (interim), self-collection (approval processes)<br>**B) General practice:** explaining delayed start age and increased interval to women, co-testing versus asymptomatic, self-collection eligibility and accuracy, sample collection, reminder and referral systems changes, education /information/communication, screening for other STIs<br>**C) Colposcopists/specialists:** work load, workforce, training/education/communication, high volumes of referral and inequity in waiting time, implications of HPV testing<br>**D) Program:** Screening register, privacy/confidentiality, privatisation of the register,

Implementation outcomes	Level of analysis	Theoretical basis	Definition	Other terms in the literature	Implementation stage	Key research question(s)	Anticipated themes & subthemes
						can you tell me about your experiences?<br><br>5) Do you think there are any other factors that will be barriers to successful implementation?	data ownership, associated costs, funding model, role change, education /information/communication
Fidelity	Individual provider	RE-AIM: part of "implementation"	*Degree to which an intervention was implemented as it was prescribed in the original protocol or as it was intended by the program developers*	Delivered as intended, adherence, integrity, quality of program delivery	Early (initial implementation) to mid	1) Do you think the renewed program has been successfully implemented so far (or as intended)?<br><br>2) Is there anything that you think should or could have been done differently, in terms of program implementation?	**BARRIERS**<br>-Delay in setting up the national register<br>-Delay in availability and resources around self-collection<br>-Education and resource availability<br>**WAYS TO OVERCOME BARRIERS**<br>-Timely availability of resources, materials to educate provider and patients<br>-National register ready in time<br>-Information and education around self-collection<br>-TGA approval for device/laboratory test for self-collection<br>-Educating women about the changes

Implementation outcomes	Level of analysis	Theoretical basis	Definition	Other terms in the literature	Implementation stage	Key research question(s)	Anticipated themes & subthemes
							-Colposcopy work force preparation/training/clinics
Feasibility	Individual provider, organisation or settings	Rogers: “compatibility” and “trialability”	*Extent to which a new treatment, or an innovation, can be successfully used or carried out within a given agency or setting*	Actual fit or utility; suitability for everyday use, practicability	Early (during adoption)	1) Overall, what do you think are the factors that will enable successful implementation of the renewal program?<br><br>2) Overall, what would you say is **the most important factor** in ensuring the successful implementation of the program? Why is that?	**FACILITATORS (MOST CRUCIAL)**<br>New screening test: more sensitive, objective and safe, tests for the cause, more robust than Pap test in the post-vaccination era<br>Triage test and age restriction: improve specificity of screening program<br>Liquid based sample: reflex cytology, no visit required<br>Invitations: reminding women before they are overdue<br>Self-collection: overcomes barriers and improves participation (high risk population)<br>Risk stratification and better management: HPV based<br>Communications tailored to community needs

Implementation outcomes	Level of analysis	Theoretical basis	Definition	Other terms in the literature	Implementation stage	Key research question(s)	Anticipated themes & subthemes
Appropriateness	Individual provider, Individual consumer, organisation or settings	Rogers: "compatibility"	**Perceived fit, relevance, or compatibility of the innovation or evidence-based practice for a given practice setting, provider or consumer; and/or perceived fit of the innovation to address a particular issue or problem. *conceptually similar to acceptability*	Perceived fit, relevance, compatibility, suitability, usefulness, practicability	Early (prior to adoption)	1) At a high level, what is your opinion of the changes in the renewed program?<br><br>2) PROBE [What do you think are the implications of the changes in the renewed program?]	**<u>ACCEPTABILITY & BARRIERS (AT AN INDIVIDUAL LEVEL)</u>**<br>A) Delayed starting age for screening (25+ years)<br>B) Increased screening interval (5 years)<br>C) New screening test & sample collection (liquid based sample & HPV based)<br>D) Self-collection (new pathway)<br>E) New referral and management pathways<br>F) New national register<br>G) Colposcopy reporting<br>H) Co-testing versus screening
Implementation cost	Provider or providing institution	TCU Program Change Model: "costs" and "resources"	*Cost impact of an implementation effort*	Cost-effective, cost-benefit, marginal cost	**Early for adoption and feasibility**<br>Mid for penetration<br>Late for sustainability	1) Has implementing the new program had any cost impacts, either positive or negative, on you/your	Program overall is cost saving to government compared to previous program but costs impact groups differently<br>-Different cost of tests and different volume fluctuations: implications for lab viability, rebates, out of pocket costs<br>-Costs associated with changing systems: practice

Implementation outcomes	Level of analysis	Theoretical basis	Definition	Other terms in the literature	Implementation stage	Key research question(s)	Anticipated themes & subthemes
						organisation? In what way?<br>2) Do you think the program implementation has been sufficiently supported by government funding? Why/why not?	systems, lab systems, IT updates and changes, register changes, redundancies<br>-Different target age group and frequency: perception of cost cutting and women <25 have to pay
Penetration	Organisation or setting	RE-AIM: necessary for "reach"	*Integration of a practice within a service setting and its subsystems; number of eligible person who use a service, divided by the total number of persons eligible for the service; number of providers who deliver a given service or treatment, divided by the total*	Spread, reach, service access, level of institutionalization	Mid to late	PARTLY CAPTURED BY THE QUESTIONS ABOVE	OVERLAPPING THEMES

Implementation outcomes	Level of analysis	Theoretical basis	Definition	Other terms in the literature	Implementation stage	Key research question(s)	Anticipated themes & subthemes
			number of providers trained in or expected to deliver the service				
Sustainability	Administrators, Organisation or setting	RE-AIM: "maintenance" Rogers: "confirmation"	*The extent to which a newly implemented treatment is maintained or institutionalized within a service setting's ongoing, stable operations*	Maintenance, continuation, durability, integration, incorporation, institutionalization, sustained use; routine	Late (long term)	WILL NOT BE CAPTURED WITHIN THE STUDY TIME PERIOD	NA
